# Satisfaction with service coverage and drug list may influence patients’ acceptance of general practitioner contract service: a cross-sectional study in Guangdong, China

**DOI:** 10.1186/s12913-019-4053-x

**Published:** 2019-04-24

**Authors:** Aiyun Chen, Shanshan Feng, Wenxi Tang, Liang Zhang

**Affiliations:** 1 Research Centre of Rural Healthcare Services, School of Medicine and Health Management, Tongji Medical College, Huazhong University of Science & Technology, No.13 Hangkong Road, Wuhan, 430030 Hubei People’s Republic of China; 2 School of Health Management, Guangzhou Medical University, Xinzao Town, Panyu District, Guangzhou, 511436 Guangdong People’s Republic of China; 3 School of International pharmaceutical Business, China Pharmaceutical University, No.639 Longmian Str, Jiangning District, Nanjing, 211198 Jiangsu People’s Republic of China

**Keywords:** General practitioner contract, Service coverage, Drug list, China

## Abstract

**Background:**

General practitioner (GP) system is proved to be effective in over 50 countries worldwide. Guangdong province, as a reform pilot in China, initiated its patient-GP contract service reform in 2014. This study is designed to assess the patients’ acceptance of General Practitioners Contract (GPC) reform and explore its influencing factors.

**Methods:**

This survey interviewed 1010 participants from 16 primary health centers (PHCs) chosen from 4 pilot cities in Guangdong during July and December in 2015. Data were collected through face-to-face interviews. The questionnaire was developed to discover the acceptance of GPC and covered three parts: respondents’ socio-demographic characteristics, health service utilization, and the patients’ assessment of primary health care centers. A binary logistic regression model was used to measure the influencing factors of respondents’ acceptance of GPC policy.

**Results:**

A total of 611(60.5%) participants accepted GPC policy. Compared to patients visited PHCs over 7 times in the previous year, those visited PHCs fewer times reported lower acceptance of GPC policy (OR:0.68, 95% CI:0.49–0.96 for visits ≤3 times and OR:0.57, 95% CI:0.38–0.84 for visits = 4–6 times). Patients’ satisfaction with medical service coverage was positively associated with patients’ acceptance of GPC (OR: 1.72, 95% CI:1.01–3.98 for the satisfied versus the dissatisfied; OR: 1.38, 95% CI:0.92–3.30 for neutral versus the dissatisfied), and the satisfaction with drug list also positively influenced patients’ acceptance of GPC policy (OR: 1.44, 95% CI:1.26–2.73 for the satisfied versus the dissatisfied; OR:1.61, 95% CI:1.36–2.99 for neutral versus the dissatisfied). Meanwhile, age and education had positive impacts on the acceptance of the GPC policy.

**Conclusion:**

This study finds out that patients’ satisfaction with medical service coverage and drug list are the influencing factors for the acceptance of GPC policy. Therefore, improvement of medical service accessibility such as better follow-up of patients with chronic diseases and enhanced referral service, as well as the expansion of drug list, will improve patients’ acceptance of GPC policy. It also finds that patients using more primary health service are inclined to accept GPC policy, so more attractive and high-quality service should be provided in PHCs.

**Electronic supplementary material:**

The online version of this article (10.1186/s12913-019-4053-x) contains supplementary material, which is available to authorized users.

## Background

The gap between the supply and demand of qualified primary health care (PHC) has been a common challenge for health care systems globally. Due to the fast population aging, increasing incidence of chronic non-communicable diseases, and the rocketing health expenditure, an effective health care delivery model that provides accessible and affordable services for the whole population is needed [1]. The evidence from more than 50 countries has proved that PHC provided by a fixed general practitioner (GP) was more effective in improving accessibility, reducing costs, and improving the patients’ health [2–6]. In a mature and stable primary health care system, a relatively long-term and stable relationship between patients and qualified GP teams could be restrained through a service contract with an integrated supply of public health and essential medical services. Countries such as Britain and Australia have adopted the models of a mandatory contract between GPs and patients, while some countries, including France, have utilized voluntary contract models [4, 7–9].

After China’s market-oriented economic reforms in the early 1980s, the three-tier health care system formed in the 1970s went collapsed, which caused the disorders of patients’ health-seeking behaviors and irrational use of health care resources [10–12]. After more than 20 years’ health care system reform, evidence has proved that the well-functioned three-level health care system is still an effective way to serve the large population of China’s health care system [13]. Therefore, the goal of China’s new round of health care reform is to reconstruct the health care system by emphasizing the role and function of primary health care. Since 2009, a large number of resources, including capital, human resources, and devices, have been invested in the primary care system, but the patient’s utilization of primary care service is still much lower than what the policy-makers expected [13]. To break the deadlock, the government aims to refine a PHC system based on GPs’ qualified and cost-effective care supply [14].

The pilot reform of the GP contract policy (referred to as GPC) initiated in several large political and economic centers in China since 2011. The GPs were required to provide integrated medical services and health management to patients, including disease prevention, free physical examination to the elder aged 65 and above, diagnosis and treatment of common diseases, referral services, and home care service. However, imaging and tests were not included. The GPC model was funded by a combined source of the fixed public health fee per capita (invested by the local government), the extra compensation from the medical insurance, and the patients themselves. Usually, the three parties shared evenly to make the model financially motivated, with an estimated expenditure amount of CNY 120 (converted into $19.8 of 2014 USD). However, not every pilot took on the same compensation formula, nor this funding model is formalized or written into the main policy.

Previous assessment for the pilot reform showed that the contracted GP services had a positive effect on hypertension and diabetes control and health outcomes [15–20]. However, there were still many patients who did not have trust in GPC or doubted about the model, which resulted in low contract acceptance in some places [16, 20].

Guangdong is a vital reform forefront with the largest provincial population of 109,990,000 and high economy (GDP per capita of 73,290 RMB and $11,037.7 of 2016 USD) by the end of 2016 [21]. The government has increased financial investments and promoted GP training programs in recent years. Though these movements had brought a significant increase in qualified human resources, merely a small increase in patient’s services utilization was observed [14]. To break the deadlock, Guangdong chose to involve in the national pilot reform of GPC policy in 11 cities and rural towns in 2014. A contract between patients and GP was made with the aims of guiding patients to make use of essential medical and public health services provided by PHCs, among which the patients’ acceptance of primary GPC policy is particularly critical.

This study is designed to explore the patients’ acceptance of GPC policy and its influencing factors. It will promote the progress of GPC policy in Guangdong, ultimately enabling the patients to make better use of services in PHCs in Guangdong.

## Methods

### Study design and sample

There were 21 cities in Guangdong. Nine of them are located alongside the Pearl River Delta and 12 cities in the mountain or coastal areas. Eight cities in Pearl River Delta and 3 cities in other regions initiated the pilot by the time the survey started. We chose 4 cities from the 11 pilot cities based on a comprehensive consideration of economic development and geographical distribution: Guangzhou, Jiangmen, Huizhou, and Chaozhou. Guangzhou, Huizhou, and Jiangmen as representatives of developed, averaged, and weak economies located within the Pearl River basin area, respectively, and Chaozhou as the wings out of the Pearl River.

PHCs in each chosen pilot city were divided into 4 groups according to the size of the covered population and performance, and one PHC was chosen to be surveyed in each group. A total of 16 facilities were included in the study. The sample size was calculated derived from a previous study about patients’ assessment of PHCs [22]. The minimum sample size of this study was estimated as 900 with a 99% confidence interval and a power of 80%. The patient interviewees were randomly chosen from each PHC, and the number was set to be 70. A total of 1120 patients participated in the questionnaire.

The questionnaire was designed by our team based on a literature study and expert opinions [16, 18, 20, 23]. The questionnaire covered: the demographic and socioeconomic characteristics, including gender, age, marital status, educational level, income level, health status, the proportion of health expenditure in total family spending, and chronic conditions. The use of PHC services, including the frequency of PHC visits in the previous year, acceptance of the GPC, and the need for PHC service content. And patient’s satisfaction towards the PHC, including 8 aspects with a Likert scale of three levels under each aspect: doctor-patient-relationship, service quality of doctors, service attitude of doctors, medical ethics of doctors, medical service coverage, medical expenditure, drug list, and medical equipment (Additional file 1).

The reliability of the evaluation scale was assessed with Cronbach’s $$ \alpha \left(\alpha =\frac{k}{k-1}\right)\cdot \left(1-\sum \begin{array}{l}k\\ {}i=1\end{array}{S_i}^2/{S_p}^2\right) $$ and the validity was assessed by the Kaiser-Meyer-Olkin (KMO) test. The reliability and validity were high (Cronbach’s *α* = 0.87 and KMO = 0.86).

### Data collection

The data were collected from July to December 2015. Before the formal investigation, a pre-survey was conducted to 40 patients from Guangzhou and Huizhou to test the feasibility of the questionnaire. Students from the School of Health Management of Guangzhou Medical University were recruited as interviewers. A one-week training was given to train the investigators the survey skills and explain the meaning of each item in the questionnaire. All participants were given face-to-face interviews, and the purpose of the study was explained to them by the interviewers.

The questionnaire completed mainly by the respondents themselves, and items and response choices were read to participants who had difficulty in reading (because of illiteracy or poor vision). The time to complete a questionnaire was 8 to 12 min. A valid questionnaire was defined as having < 5% missing data.

### Data processing and statistical analysis

Data were entered into the database by two analysts independently. Patients’ acceptance of GPC policy was assessed by a single question“Did you ever sign a contract with GP?” Chi-squared (χ2) test was used to compare the sociodemographic characteristics, medical service utilization of PHCs and patients’ satisfaction in two groups (acceptable vs. non-acceptable). Binary logistic regression analysis was used to measure the effect of patients’ satisfaction and primary care service utilization on patients’ acceptance of GPC policy. A three-scale grading system was used to measure patients’ satisfaction: satisfied, neutral and dissatisfied. Medical service utilization of PHCs was measured by the number of visits in PHCs in the previous year coded as≤3 times, 4–6 times and ≥ 7 times. The stepwise selection method was used to include/exclude variables in/from the logistic regression model. PASW Statistics 18.0 was used, and the statistical significance level was set at 0.05.

## Results

A total of 1120 patients received interviews, and 1010 valid questionnaires were collected. The overall response rate was 90.18%.

### Patients’ acceptance of GPC policy and distribution

Six hundred eleven participants (60.5%) accepted the GPC policy. Table 1 reported the socio-demographic characteristics and health service utilization of the participants. No significant difference was found in gender, marital status, self-reported health status or chronic conditions between groups who accepted to General practitioner contract policy (AGPC) and who didn’t (NAGPC), but significant differences were found in age, education background, monthly family income, proportion of medical expenditure to total family expenditures, and frequency of visiting PHCs.

**Table 1:** The characteristics of respondents and health service utilization according to acceptance

	AGPC		NAGPC		x^2^	*P*-value
	n(611)	%	n(399)	%		
Gender						
Male	262	42.88	150	37.59	2.79	0.10
Female	349	57.12	249	62.41		
Age						
18–34	28	4.58	44	11.03	28.49	< 0.01
35–49	68	11.13	55	13.78		
50–64	190	31.10	146	36.59		
≥ 65	325	53.19	154	38.60		
Marital status						
Married	517	84.62	323	80.95	2.31	0.13
Unmarried (single/divorced/windowed)	94	15.38	76	19.05		
Education background						
Primary school and below	335	54.83	157	39.35	26.28	< 0.01
Middle school	238	38.95	195	48.87		
College degree and above	38	6.22	47	11.78		
Income of family monthly (of 2014 USD)						
Under $495.9	469	76.76	237	59.40	34.58	< 0.01
$495.9–$1157.0	118	19.31	135	33.83		
≥$1157.0	24	3.93	27	6.77		
Self-reported health status						
Well	222	36.33	152	38.10	2.22	0.33
General	270	44.19	184	46.11		
Bad	119	19.48	63	15.79		
Chronic conditions						
Yes	274	44.84	188	47.12	0.44	0.51
No	337	55.16	211	52.88		
The proportion of medical expenditure to total family expenditures						
< 10%	306	50.08	162	40.60	8.73	0.01
10–29%	217	35.52	168	42.11		
≥30%	88	14.40	69	17.29		
Number of visiting PHCs						
≤3	267	43.70	161	40.35	7.25	0.03
4-6	143	23.40	75	18.80		
≥7	201	32.90	163	40.85		

Patients’ satisfaction assessment of PHCs

The differences of satisfaction showed in each aspect between AGPC and NAGPC were significant, with a higher satisfaction rate in the group of AGPC (as showed in Table 2).

**Table 2:** Distribution of participants’ satisfaction according to acceptance

	AGPC		NAGPC		x^2^	*P*-value
	n(611)	%	n(399)	%		
Doctor-patient-relationship						
Satisfied	462	75.61	273	68.42	7.48	0.02
Neutral	130	21.28	115	28.82		
Dissatisfied	19	3.11	11	2.76		
Service quality of doctors						
Satisfied	493	80.68	273	68.42	23.91	< 0.01
Neutral	86	14.08	105	26.32		
Dissatisfied	32	5.24	21	5.26		
Service attitude of doctors						
Satisfied	547	89.53	323	80.95	14.87	0.01
Neutral	50	8.18	60	15.04		
Dissatisfied	14	2.29	16	4.01		
Medical ethics of doctors						
Satisfied	554	90.67	327	81.95	16.51	< 0.01
Neutral	45	7.37	58	14.54		
Dissatisfied	12	1.96	14	3.51		
Medical service coverage						
Satisfied	510	83.47	271	67.92	41.96	< 0.01
Neutral	63	10.31	102	25.56		
Dissatisfied	38	6.22	26	6.52		
Medical expenditure						
Satisfied	444	72.67	250	62.66	11.34	< 0.01
Neutral	122	19.97	111	27.82		
Dissatisfied	45	7.36	38	9.52		
Drug list						
Satisfied	393	64.33	162	40.61	57.85	< 0.01
Neutral	124	20.29	116	29.07		
Dissatisfied	94	15.38	121	30.32		
Medical equipment						
Satisfied	373	61.05	157	39.34	46.87	< 0.01
Neutral	161	26.35	152	38.10		
Dissatisfied	77	12.60	90	22.56		

### Influencing factors for participants’ acceptance of GPC policy

Binary logistic regression was performed to explore the influencing factors for acceptance of GPC policy, and the results were presented in Table 3. After controlled for the cofounders, compared to patients who visited PHCs over 7 times the previous year, patients who visited PHCs less than three times or four to six times were associated with lower acceptance (OR:0.68, 95% CI: 0.49–0.96 for visits≤3 times and OR:0.57, 95% CI: 0.38–0.84 for visits = 4–6 times). Satisfaction with medical service coverage affected patients’ acceptance of GPC (OR:1.72, 95% CI: 1.01–3.98 for satisfied vs. dissatisfied and OR:1.38, 95% CI:0.92–3.30 for neutral vs. dissatisfied). Besides, the satisfaction with drug list also influenced patients’ acceptance (OR: 1.44, 95% CI: 1.26–2.73 for satisfied vs. dissatisfied and OR: 1.61, 95% CI:1.36–2.99 for neutral vs. dissatisfied). Furthermore, age was an influencing factor for patients’ acceptance of GPC, with the older people the higher acceptance probability (OR:0.73, 95% CI: 0.39–1.76 for 18–34 vs. ≥65 group; OR:0.69, 95% CI: 0.38–1.18 for 35–49 vs. ≥65 group). People with higher education level was associated with greater acceptance (OR: 0.51, 95% CI: 0.30–1.25 for primary school and below vs. college degree and above).

**Table 3:** Factors affecting acceptance of General Practitioner among participants using binary logistic regression

Variables	OR	95% CI	*P*-value
Number of visits to PHC (ref. = 7 times and above)			
≤ 3	0.68	0.49–0.96	0.03
4-6	0.57	0.38–0.84	< 0.01
Doctor-patient-relationship^a^			
Satisfied	1.22	0.94–2.89	0.66
Neutral	1.39	0.88–2.72	0.48
Service quality of doctors^a^			
Satisfied	1.33	0.84–2.97	0.48
Neutral	1.52	1.05–3.37	0.31
Service attitude of doctors^a^			
Satisfied	1.50	1.14–2.89	0.31
Neutral	1.52	1.14–2.99	0.34
Medical ethics of doctors^a^			
Satisfied	1.76	1.26–3.14	0.76
Neutral	1.89	1.18–3.16	0.89
Medical service coverage^a^			
Satisfied	1.72	1.01–3.98	0.01
Neutral	1.38	0.92–3.30	< 0.01
Medical expenditure^a^			
Satisfied	0.81	0.41–1.62	0.55
Neutral	0.73	0.36–1.49	0.39
Drug list^a^			
Satisfied	1.44	1.26–2.73	< 0.01
Neutral	1.61	1.36–2.99	0.04
Medical equipment^a^			
Satisfied	1.77	1.03–3.53	0.18
Neutral	0.95	0.53–1.69	0.86
Gender(ref. = Female)			
Male	1.22	0.91–1.63	0.18
Age(ref. = more than 65)			
18–34	0.73	0.39–1.76	< 0.01
35–49	0.69	0.38–1.18	0.02
50–64	0.85	0.59–1.80	0.06
Marital status(ref. = unmarried)			
Married	1.40	0.94–2.01	0.10
Education background (ref. = College degree and above)			
Primary school and below	0.51	0.30–1.25	0.01
Middle school	0.75	0.48–1.24	0.26
Income of family monthly (ref. = $1157.0 and above)			
Under $495.9	0.64	0.34–1.21	0.17
$495.9–$1157.0	1.33	0.69–2.59	0.39
Proportion of medical expenditure to total family expenditure(ref. = 30% and above)			
< 10%	0.69	0.44–1.07	0.10
10–29%	0.80	0.52–1.24	0.32
Self-reported health status (ref. = bad)			
Well	1.33	0.85–2.01	0.22
General	1.46	0.97–2.22	0.07
Chronic conditions(ref. = No)			
Yes	1.03	0.86–1.78	0.30

^a^the reference group was the dissatisfied group

## Discussion

Our finding showed that the overall proportion of patients who accept the GPC policy was 60.5% in Guangdong, which is higher than Shanghai of 52.8% in 2013 [16], and 51.5% in Beijing 2012 [17].

The results of the binary regression analysis suggested that the patients’ visit frequency to PHCs was an influencing factor. Patients who made more use of health service in PHCs showed a stronger willingness to accept GPC policy. Since China’s 2009 health care system reform, various primary care services have been provided in the public health package, such as free physical examination for patients aged 65 years and above, medication consultation, and health education. Physical examination and medication consultation were most frequently used by the elderly. Therefore, patients aged 65 and above, and patients who visited PHCs more frequently were more inclined to accept GPC policy. This trend also meant that patients’ acceptance of GPC service was related to public health service provided by PHCs, which was also consistent with the policy priority setting to serve the aged people.

It also found that patients’ assessment on PHCs was also associated with acceptance of GPC policy. When the participants were asked “why did not you sign a contract with GP,” the most frequent reason was “I am not satisfied with the PHCs.” By analysis patient satisfaction from eight dimensions, the results showed that the participants gave a good score on service attitude and medical ethics of doctors, but a poor rate on medical expenditure, the limited drugs in the drug list and medical equipment provided in PHCs. However, there were only two dimensions, including medical service coverage and satisfaction of the drug list, had a significant impact on acceptance. The previous research which used a EUROPEP scale showed that medical care and organization of care were two dimensions influencing patients’ acceptance of gatekeeper policy in Wuhan, China [23]. Despite differences in survey tools, the conclusion in the medical care coverage had similarity. There were also studies pointing out that patients had a poor assessment of drug list in PHCs in China [24]. Therefore, medical service coverage and drug list were of great importance in improving PHCs’ service and patients’ satisfaction.

In the new round of Chinese medical system reform, a hierarchical health care system was put forward, and PHCs was designed as the patient’s first contact with the whole system. However, the medical service coverage that could be offered in PHCs were limited at this stage. When the participants were asked, “What kind of medical service do you need most?” Medical consultation, follow-up of chronic diseases, and referral services were the top three answers. This finding was in accordance with previous studies [16, 19, 20, 25]. There are supposedly two ways to address this issue. First, to publicize the patients about the functions of PHCs, and offer them the most frequently needed medical services. Secondly, to provide a coordinated referral service to satisfy patients’ medical needs. When the patient’s disease cannot be solved in the PHCs, they should be referred to the upper-level hospital conveniently.

Drug list provided in PHCs is another factor influencing patients’ acceptance of GPC. The national system for essential drugs at the primary level was implemented since 2009. Only 307 kinds of drugs, including chemicals and herbal medicine, could be used in PHCs. When patients who were reluctant to accept the GPC were asked about that “What disappointed you most about PHCs?” the majority of them replied with “I cannot get all the medicine I need. And when medicines are prescribed in PHCs, I have to go to a tertiary hospital to get them.” Therefore, national essential drug list should be expanded, and measures such as increasing the use of non-essential drugs at the grassroots level should be taken. Some cities have conducted pilot reforms, but the effect needs further verification [24, 26, 27].

There are several limitations in this research. First, the potential influencing factors of patients’ acceptance of policy are possibly more than what we investigated in this research. Second, the investigation was conducted from the demand side. The data we collected were recalled by the respondents within 12 months. Given the frequency of PHC use, we have to stay at this length of a period. Otherwise, there could be a higher probability of fewer differences among all the respondents. Therefore, recall bias could be a potential limitation that reduces the reliability in our analysis. There is no good way to solve this problem. Nevertheless, we tried to control the bias to our great ability. During the survey, we trained our investigator to improve their investigation skills, encouraging them to help respondents recalling by treasuring their patience. Furthermore, there are also studies indicated that some bias might not be as significant as we thought, because the missing information neglected by the respondents might not be so important as to recognize [28].

In the following studies, we will also investigate other stakeholders, including the suppliers and regulators, to form a complete chain of evidence and promote the popularization of GP in Guangdong and other similar areas in China.

## Conclusions

GPC policy is a significant reform to strengthen the utilization of primary health service and form a strong hierarchical medical delivery pattern. The patients’ acceptance is the key to this policy. Our study suggests that improvement of medical service accessibility such as follow-up of chronic diseases and referral service, as well as the expansion of drug list, will improve patients’ acceptance of GPC policy. This study also shows that patients who make more use of primary health service are inclined to accept the GPC service, so more attractive and high-quality services should be provided by PHCs.

## Additional files

Additional file 1: Questionnaire. (DOCX 26 kb)

Additional file 2: Medical ethics approval. (DOCX 16 kb)

Additional file 3: Informed consent. (DOC 35 kb)

## Abbreviations

AGPC: Accept to General practitioner contract
CI: Confidence Interval
GDP: Gross domestic product
GP: General practitioner
GPC: General practitioner contract
OR: Odds Ratio
PHC: Primary health centers, including community health centers, township hospitals, and village clinics
