# Supplementary material for: Satisfaction with service coverage and drug list may influence patients’ acceptance of general practitioner contract service: a cross-sectional study in Guangdong, China
Source: BMC Health Serv Res. 2019 Apr 24;19:251. doi: 10.1186/s12913-019-4053-x (PMC6480488; doi:10.1186/s12913-019-4053-x)
Supplement: Supplementary file 1 — Questionnaire. (DOCX 26 kb) [file 12913_2019_4053_MOESM1_ESM.docx]

Questionnaire No.: |___|___|___|___|___|

A Survey on the Utilization and Acceptance of General Practitioner Contract Policy in Guangdong Province

General Practitioner Contract policy is a significant health care service reform in Guangdong. We would like to learn about your utilization and acceptance of the service. This might include your personal information, your utilization of primary health service, your assessment of medical facility or doctors, and your suggestions on improving the service. Your answers will be kept confidential and will not be shared with anyone else.

Part I Personal Information

1. Gender： 🞎male 🞎female

2. Age(year)：

3. Marital status： 🞎married 🞎unmarried（single/divorced/widowed）

4. Education background：🞎primary school and below 🞎middle school 🞎college degree or above

5. The average income of my family monthly in the past year was:

🞎under $495.9 🞎$495.9-$1157.0 🞎 $1157.0 and above

6. The proportion of medical expenditure to total family expenditure last year was:

🞎less than 10% 🞎10-29% 🞎30% or above

7. My health status last year was: 🞎well 🞎general 🞎bad

8. I had diagnosed with chronic diseases.

🞎yes 🞎no

Part Ⅱ Utilization of Primary Health Service

1. When I feel discomfort, my preferred medical facility is:

🞎community health service center 🞎village or private clinic 🞎township hospital 🞎district or above hospital

2. The number of my visiting the community health service center /village clinic in the previous year was:

🞎less than 3 times 🞎4-6 times 🞎7 times and above

3. The payment of my medical expenditure is:

🞎pay in full 🞎partly by health insurance

4. If I walk to the nearest community health service center /village clinic, the time it will take is:

🞎less than 15 minutes 🞎16-29 minutes 🞎more than 30 minutes

Please comment on the community health service center /village clinic you usually visited.

5.

5.1 My assessment of the doctor-patient relationship is:

🞎satisfied 🞎neutral 🞎dissatisfied

5.2 My assessment of the service quality of doctors is:

🞎satisfied 🞎neutral 🞎dissatisfied

5.3 My assessment of the service attitude of doctors is:

🞎satisfied 🞎neutral 🞎dissatisfied

5.4 My assessment of the medical ethics of doctors is:

🞎satisfied 🞎neutral 🞎dissatisfied

5.5 My assessment of medical service coverage is:

🞎satisfied 🞎neutral 🞎dissatisfied

5.6 My assessment of medical expenditure is:

🞎satisfied 🞎neutral 🞎dissatisfied

5.7 My assessment of the drug list is:

🞎satisfied 🞎neutral 🞎dissatisfied

5.8 My assessment of the medical equipment is:

🞎satisfied 🞎neutral 🞎dissatisfied

Part Ⅲ A Survey on Utilization & Needs of Family Doctor Service

1. My knowledge about General Practitioner Contract Policy is:

🞎well 🞎 a little 🞎nothing

2. I signed a contract with GP.

🞎yes 🞎 no（Please go to 2.2）

2.1 The reasons for my signing the contract are（multiple choices are allowed）：

🞎It will be more convenient to see a doctor and get medicines.

🞎I will pay less for medical service.

🞎I can get medical/health consultation from GPs.

🞎I can get better follow-up service of chronic disease.

🞎I can get home care service.

🞎I can get a priority appointment.

🞎I can get referral services.

🞎Others. Please list.

(Please go to 3.)

2.2 The reasons for not signing the contract are（multiple choices are allowed）：

🞎I don’t know about the policy exactly.

🞎I’m afraid of restricting my choice to see a doctor.

🞎I’m not satisfied with the service provided by the community health service center /village clinic.

🞎I’m not satisfied with the drug list, and I can’t get the medicine I need.

🞎I prefer to go to see a doctor in tertiary hospitals.

🞎Others. Please list.

1. The medical services I need most are: (multiple choices are allowed.)

🞎outpatient service

🞎medical consultation

🞎home care services

🞎health education

🞎follow-up of chronic diseases

🞎referral services

🞎Others. Please list.

1. I have some suggestions for improving the General Practitioner Contract Policy.

🞎No

🞎Yes. Please list.

investigator：

date：
