# Supplementary material for: Satisfaction with service coverage and drug list may influence patients’ acceptance of general practitioner contract service: a cross-sectional study in Guangdong, China
Source: BMC Health Serv Res. 2019 Apr 24;19:251. doi: 10.1186/s12913-019-4053-x (PMC6480488; doi:10.1186/s12913-019-4053-x)
Supplement: Supplementary file 2 — Medical ethics approval. (DOCX 16 kb) [file 12913_2019_4053_MOESM2_ESM.docx]

**Medical Ethics Approval of Guangzhou Medical University**

**Serial number：20150416**

| Project name | Evaluation of General Practitioner Contract Policy in Guangdong Province | | | |
| --- | --- | --- | --- | --- |
| Research contents | General Practitioner Contract Policy is a significant health care service reform in China. This study aims to survey the utilization and assessment of primary health service and patients’ acceptance of the General Practitioner Contract Policy. | | | |
| Reviewed documents | Medical ethics application Informed consent | | | |
| Researcher | Feng Shanshan, Associate professor | | | |
| Ethics committee members | Name | Title | Major | Employer |
|  | Zhang Yajie | Professor | pathology | School of Preclinical Medicine, Guangzhou Medical University |
|  | Wang Jian | Professor | Internal Medicine | The First Affiliated Hospital of Guangzhou Medical University |
|  | Liu Junrong | Professor | Ethics | School of Health Management, Guangzhou Medical University |
|  | Ma Jinxiang | Associate professor | Statistics | School of Public Health, Guangzhou Medical University |
|  | Shang Herui | Professor | psychology | School of Health Management, Guangzhou Medical University |
|  | Xiao Peng | Associate professor | Law | School of Health Management, Guangzhou Medical University |
|  | Zhou Meifang | Associate professor | Public Affairs Administration | School of Health Management, Guangzhou Medical University |
| The ethics committee comments | According to the Regulations on the Ethical Review of Biomedical Research Involving Human Beings enacted by the Health Department of the People's Republic of China, the Helsinki declaration and other relevant documents, the decision of the ethics committee is following:  🗹Agree □Modification □Reviewed after modification □Disagree  (note: if the decision of reviewed after modification or disagree is made, please give the reasons.) | | | |
| Signature of the committee director: Zhang Yajie Date: April 16, 2015 | | | | |
