# Supplementary material for: Satisfaction with service coverage and drug list may influence patients’ acceptance of general practitioner contract service: a cross-sectional study in Guangdong, China
Source: BMC Health Serv Res. 2019 Apr 24;19:251. doi: 10.1186/s12913-019-4053-x (PMC6480488; doi:10.1186/s12913-019-4053-x)
Supplement: Supplementary file 3 — Informed consent. (DOC 35 kb) [file 12913_2019_4053_MOESM3_ESM.doc]

Informed Consent

Dear Madam / Sir,

You will be invited to participate in the survey on Evaluation of General Practitioner Contract Policy in Guangdong Province, which has been reviewed by the Ethics Committee of Guangzhou Medical University. This informed consent provides detailed information to help you decide whether or not to participate in this project. Please read it carefully.

We would like to learn about the utilization and acceptance of General Practitioner Contract Policy. The data we collect might include your personal information, the utilization of primary health service, your assessment of medical facility or doctors, and your suggestions on improving the primary care service.

It may bring some risks and discomforts in the survey, as personal information and the evaluation of primary health services are required. However, your participation will help medical facilities to provide better health care service and ultimately benefit yourself.

The researcher will afford all the expenses for this project. If you suffer from any research-related injury, we will provide compensation and available medical treatment.

As a respondent, you are required to provide the data such as personal information, your health status, your medical seeking behavior in primary health care facilities, your evaluation of primary health care, and acceptance of General Practitioner Contract Policy. It may take about ten minutes.

If you decide to participate in this research, your data will be kept confidential and will not be shared with anyone else. Your personal information will not be disclosed when the results of our study are published.

Your participation in this research is entirely voluntary. Refusal to participate or to discontinue participation at any time will involve no penalty or loss of benefits.

If you have any questions, please don’t hesitate to contact the researcher.

Inform statement of the researcher

I confirm that I have explained the project details to the subject, including his/her rights and possible benefits and risks.

Signature of the researcher: Feng Shanshan Tel: 13302292195 Date April 16, 2015

Consent statement of the subject

I have carefully read the above introduction to this project. I understand and accept the potential risks and benefits. I acknowledge that participating in the study is entirely voluntary.

Signature of the subject Contact information: Date:

Signature of the subject agent Contact information: Date:

**(**Note: If the subject is illiterate, the signature of the witness is required; if the subject is incapacitated, the signature of the agent is required.**)**
